# A Case of Concussion of the Brain, with Remarkable Recovery

**Published:** 1840-06

**Authors:** J. L. Burtt

**Affiliations:** Cincinnati, Ohio


Art. V.—*A Case of Concussion of the Brain.* By J. L. Burtt, M. D., of Cincinnati, Ohio.

May 27th, 1840, the mother being absent, the child which forms the subject of the present paper, aged three years, was left in charge of some of the rest of the family, and was suffered to climb over the sill of a window in the second story of a large dwelling house. From thence, a height of perhaps 16 feet, it fell upon the brick pavement beneath. Falling upon the vertex of the head, when taken up, and for an hour afterwards, the cranial bones were so depressed as to present an almost level surface. I might safely say, that its head was as flat as a table.

The *os frontis* projected forward at least two inches over the eye brows, in its natural position. The child was examined by Drs. Mussey and Fore. It presented all the symptoms of violent concussion of the brain for about an hour.

Its head was shaved, but on examination no symptom of fracture was found as had been expected. After this period, a spasmodic action of the facial muscles ensued, and shortly after it vomited several times, which seemed to relieve it and in a great degree to overcome the remaining stupor.

Cold applications to the head, and enemata, which were speedily followed by copious evacuations, formed the treatment at this period. From the time that its bowels acted it seemed perfectly sensible, knowing its mother and asking for water. In the mean time, the cranium had made considerable advances towards its proper elevation. I sat up with it during the night—it rested well; the reaction was not high, so that bleeding was not necessary; and the cranial bones rapidly assumed their natural position.

From this time until the 30th, the convalescence was rapid, the only treatment being cold applications to the head, and the administration of gentle cathartics.

31st. Patient able to walk, with a little assistance.

June 2d. To all appearance perfectly well.

*Cincinnati*, June, 1840.

